# Explainable Anomaly Detection Framework for Maritime Main Engine Sensor Data

**DOI:** 10.3390/s21155200

**Published:** 2021-07-31

**Authors:** Donghyun Kim, Gian Antariksa, Melia Putri Handayani, Sangbong Lee, Jihwan Lee

**Affiliations:** 1 Korea Marine Equipment Research Institute, Busan 49111, Korea; kimdonghyun9942@gmail.com; 2 Department of Industrial and Data Engineering, Major in Industrial Data Science and Engineering, Pukyong National University, Busan 48513, Korea; gian.antariksa@gmail.com (G.A.); meliaph@pukyong.ac.kr (M.P.H.); 3 Lab021 Shipping Analytics, Busan 48508, Korea; sblee@lab021.co.kr

**Keywords:** explainable AI, anomaly detection, isolation forest, Shapley Additive exPlanations, SHAP, clustering, marine engine, onboard sensors

## Abstract

In this study, we proposed a data-driven approach to the condition monitoring of the marine engine. Although several unsupervised methods in the maritime industry have existed, the common limitation was the interpretation of the anomaly; they do not explain why the model classifies specific data instances as an anomaly. This study combines explainable AI techniques with anomaly detection algorithm to overcome the limitation above. As an explainable AI method, this study adopts Shapley Additive exPlanations (SHAP), which is theoretically solid and compatible with any kind of machine learning algorithm. SHAP enables us to measure the marginal contribution of each sensor variable to an anomaly. Thus, one can easily specify which sensor is responsible for the specific anomaly. To illustrate our framework, the actual sensor stream obtained from the cargo vessel collected over 10 months was analyzed. In this analysis, we performed hierarchical clustering analysis with transformed SHAP values to interpret and group common anomaly patterns. We showed that anomaly interpretation and segmentation using SHAP value provides more useful interpretation compared to the case without using SHAP value.

## 1. Introduction

Modern vessels utilize onboard sensors and data acquisition systems to collect ship performance and navigation parameters [1]. The explosion of the sensor data enables one to apply machine learning techniques to the data to extract knowledge through data analysis [2] and even trigger interesting data-driven applications, including fuel consumption prediction [3,4], prediction of speed loss [5], propulsion power prediction [6], weather routing [7], development of monitoring systems [8] and enhanced inspection [9], energy efficiency analysis [10], trim optimization [11], predictive maintenance [12], data-driven prognostics [13].

### 1.1. Data-Driven Approach to Condition-Based Monitoring of Marine Engines

One of the promising data-driven approaches using vessel datasets is condition monitoring of the vessel main engine. The main engine is the most critical subsystem in a vessel because a failure of the engine may lead to severe accidents, endangering crew and passengers onboard, posing a threat to the environment, damaging the ship itself, and incurring a significant business loss [14]. Thus, several researchers tried to analyze the essential sensor signals related to the main engine, such as power, speed, temperature, and pressures for selected engine components, to detect anomalous data points that may indicate critical information for the failure [15].

The ideal approach to data-driven condition monitoring would be to use the run-to-failure data obtained from the actual engine failure event. If such data is available, a classification model can be directly applied to predict the failure from the sensor data. However, it is hard to learn from the actual failure event because engine failure is an extremely rare event in practice. Instead, the artificial dataset is usually adopted in the maritime domain. For example, Kowalski et al. [16] utilized the marine engine sensor dataset obtained from the laboratory experiment. The dataset consists of 14 faulty conditions additional to normal behavior, and a machine learning model was adopted to discern failure types. Cipollini et al. [17] utilized a computer simulation model. Several sensor values were generated in their dataset according to the decay process of four-engine components (gas turbine, gas turbine compressor, hull, and propeller). Simple supervised regression models were then adopted to predict the decay coefficient given only the sensor dataset. Utilizing the same dataset, Cipollini et al. [17] and Tan et al. [18] proposed a classification-based approach and compared the accuracy of several competing classification algorithms. However, generating the simulated dataset is costly because it requires complex physical modeling of the system, which may be challenging to develop.

Unsupervised learning is another alternative when the labeled dataset is unavailable. The unsupervised approach assumes no prior information about the failure label of the dataset. Instead, it detects the anomalous point that shows a significant deviation from the rest of the dataset. It is, of course, noteworthy that such an anomalous point does not always indicate the fault or failure of the engine. However, at least, the anomalous point may indicate the sudden change of the system, which may lead to system fault for failure [16]. Several unsupervised anomaly detection algorithms have been proposed in the maritime domain to monitor the anomalous behavior in the vessel main engine. For example, Vanem and Brandsæter [19] adapted the cluster-based anomaly detection to compare the detected anomaly ratio among the implemented clustering techniques and draw the base contamination rate of the anomaly ratio results from all the compared methods. Brandsæter et al. [20] proposed another way to detect anomalies using online anomaly detection with baseline deviation modeled using the auto-associative kernel regression that constructed the representative of the normal condition. On the other hand, Bae et al. [21] proposed a statistical process control (SPC)-based approach. Bootstrap-based T2 multivariate chart proposed by Phaladiganon et al. [22] is adopted to find the threshold values of each sensor. An outlier is detected when at least one of the sensor values falls outside the threshold. However, their approach suffers from low performance when the dataset involves high dimensional spaces [23]. Recently, Kim et al. [24] proposed an ensemble-based method that is scalable to high-dimensional and large-scale sensor data. 

### 1.2. Explainable AI for Anomaly Detection 

Explaining the possible cause of anomalies is necessary for conditioned monitoring of marine engines because this additional knowledge about anomalies would provide much insight for the user to prepare for the following action. Unfortunately, the current methods do not explain the rationale behind the model’s prediction. Given an anomaly found, the user cannot easily understand which sensor is most responsible for this anomaly. Previously, Kim et al. [24] tried to explain the anomaly pattern by performing the clustering analysis with anomalous data. However, their method could not quantify the degree of contribution for each sensor on found anomalies.

To overcome this limitation, this paper adopts explainable AI (XAI) techniques to explain the maritime engine anomalies. The XAI methods aim to assess the feature attributions, which indicates how much each feature in a model contributed to the predictions for each given instance. For the simple machine learning model like linear regression or logistic regression, one can quickly evaluate the feature importance by referring to the coefficient associated with each data feature. However, it is hard for a human to measure the feature attribution for complex models such as random forest or artificial neural networks because too many parameters are involved in the model. In this regard, several XAI techniques have been proposed to evaluate feature attribution of individual predictions obtained from the complex machine learning model during past decades. One notable breakthrough was Local Interpretable Model-agnostic Explanations (LIME) proposed by [25]. LIME takes the input instance and slightly perturbs that input many times, then fits a linear model to those perturbations and produces an explanation in terms of the linear model’s coefficients. More recent work by Lundberg et al. [26] identified the relations between LIME and Shapely Value and combined them into unified framework called SHAP (Shapley Additive exPlanations) values. SHAP enables anyone using complex machine learning models to explain their model’s predictions through the lens of the input features. For more literature review about explainable AI methods, please refer to [27].

In the literature, the SHAP value was applied to explain the anomaly detection algorithm. For example, the authors of [28] explain the intrusion detection systems used in the cybersecurity system of a network. In the engineering system, SHAP was also used to explain the energy efficiency prediction of the water pumping systems [29] and the heating systems [30]. In the medical field, SHAP was used to explain cancer prediction from the tree-based anomaly detection model [31], and risk factors of hypoxemia during surgery [32]. 

### 1.3. Aim of This Study 

This study tries to explain the anomalous marine engine behavior with SHAP values. As a base anomaly detector, the isolation forest developed by Liu et al. [33] is adopted. isolation forest is an ensemble-based decision tree for anomaly detection. As with other ensemble-based methods like the random forest, isolation forest utilized no distance or density measures, and thus methodology was performed satisfactorily in high-dimensional data that contains many irrelevant attributes. After the anomaly score is obtained, SHAP was combined with the isolation forest to calculate the feature importance of individual prediction. Thus, given an anomalous data point, anyone can easily explain which sensor value is responsible for the anomaly. Additionally, we performed the clustering analysis with aggregated feature importance values to investigate the common anomaly pattern. The transformed value using SHAP was useful for the anomaly segmentation and visualization. To validate our method, we analyzed the actual marine engine dataset collected from the cargo vessel that operates around the Asian-Pacific Ocean. 

The contribution of this paper is two-fold. First, to the author’s knowledge, this is the first attempt to use explainable AI in anomaly detection in the maritime domain, particularly utilizing marine engine datasets. With our framework, one can explain the possible cause of anomalies by specifying suspicious sensors. This additional knowledge about anomaly would provide much insight for the user to prepare for the following action. Second, we performed much in-depth analysis using feature importance values obtained from SHAP. We showed that the clustering method and visualization tool provided in this study was useful for anomaly segmentation and interpretation. We also compared the usefulness of this framework by comparing the case without SHAP value.

The remainder of this paper is organized as follows. Section 2 describes brief information about the target vessel and dataset. After that, Section 3 explains the background theory about models used in this study. In Section 4, we explain our procedure and discuss the experiment result. Finally, Section 5 explains the conclusion and future direction of the study.

## 2. Data Description and Exploratory Analysis

### 2.1. Dataset

**Figure 1:**
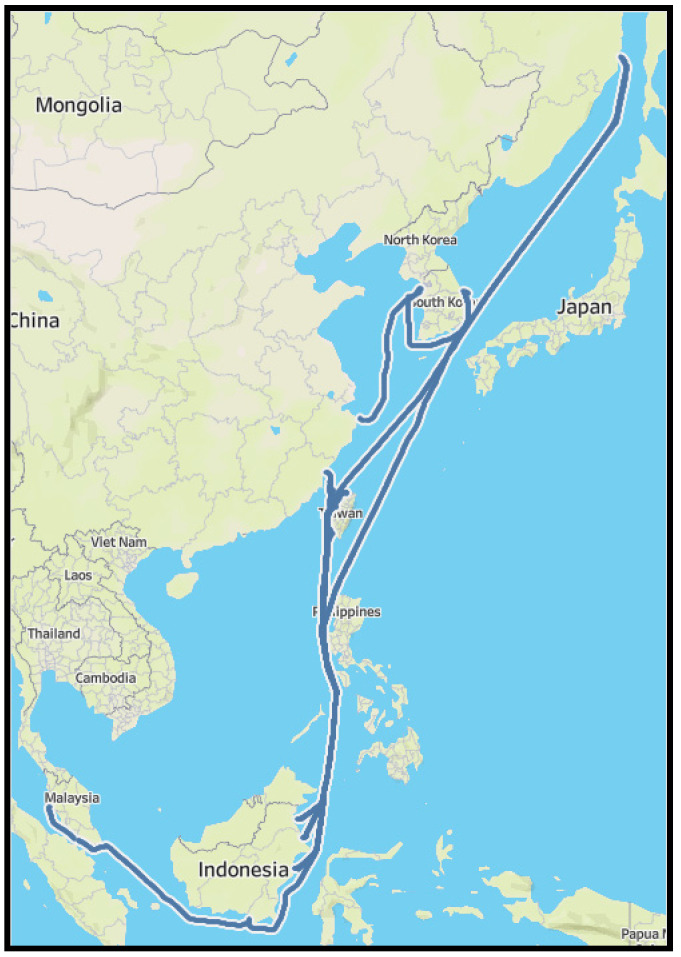
Vessel routes trajectory.

The dataset used in this research is retrieved from a 200,000-ton bulk cargo ship collected from its engines during 10 months of operation time from July 2019 to April 2020. During the data collection period, the vessel has operated around 30 voyages visiting several Asian ports, including South Korea, Russia, Taiwan, Indonesia, Malaysia, Singapore, and the Philippines. The sensor data is recorded in one second and results in a total of 22,513,800 observations. We thought that the size of the dataset is large enough for anomaly detection analysis because the target vessel operates the same route several times during the data collection period. Additionally, the dataset size was larger than the previous studies. For Cheliotis et al. [34], the data collection period was only three months. For Brandsaeter et al. [20], the data collection period was the same (around 10 months), but only 10 sensors were used for the analysis. The overall route is shown in Figure 1. The detailed specification of the vessel is shown in Table 1.

The engine type of the target vessel is MAN B&W MC50, a two-stroke engine with a moderate rpm [35]. The engine adopts variable injection timing (VIT) systems that regulate the ignition of the fuel injection. The coolant system cools the moving components (crankshaft, piston) with lubricant, while the stationary components (cylinder head, jacket) are cooled with fresh water. Seawater is used to cool the coolant in a separate heat exchanger.

### 2.2. Data Features

The original data consists of 144 different sensors installed across vessel machinery systems. In this study, the domain expert carefully chose the sensors that were related to the main engine. The list of chosen sensors is shown in Table 2.

## 3. Overall Framework and Background

### 3.1. Overall Framework

**Figure 2:**
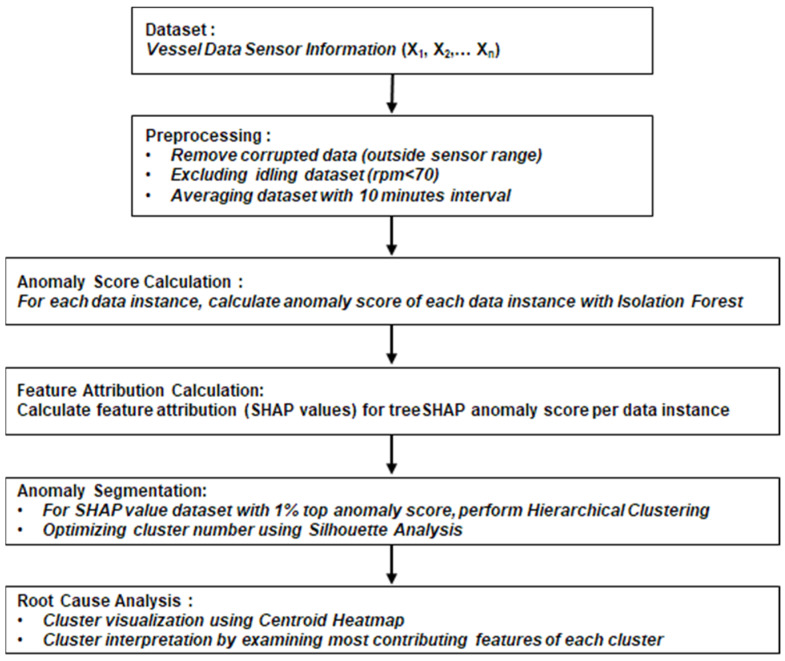
Proposed framework approach.

The whole approach for this study is depicted in Figure 2. To begin, we preprocessed the dataset to simplify the execution of unsupervised learning models. After that, anomaly detection was completed on the vessel instances to a certain suspicious condition. Isolation forest was applied to the preprocessed dataset to identify the anomalous data instance in the sensor stream as a whole. Following that, explainable AI named SHAP (Shapley Additive exPlanations) was utilized to transform the dataset into specific instances based on the explainability of the isolation forest model. As previously stated, SHAP was utilized to determine the contribution of each sensor’s features to the discovered anomaly. SHAP enables quantification of which sensor is accountable for each incident of abnormal data. This procedure interpreted the model’s local and global explanations, respectively. Finally, the segmentation of SHAP value on anomalous data points was performed using the hierarchical cluster method, then segregated into multiple clusters. After identifying clusters, we highlighted the sensors that contributed the most to the cluster.

### 3.2. Unsupervised Anomaly Detection and Isolation Forest

Unsupervised machine learning algorithms do not learn from labeled samples but rather comprehend the data structure to classify it into a fixed or variable number of classes. They are based on two fundamental assumptions. First, they assume that only a very tiny percentage of the data is anomalous. Second, the anomalous data will exhibit statistically significant differences from the normal. According to these two assumptions, data groups of similar instances that appear frequently are considered normal, but observations that appear infrequently and are significantly different from the majority of the instances are considered an anomaly.

In the literature, there have been several anomaly detection methods with a specific algorithm, such as (1) proximity-based model: local outlier factor (LOF) [36], k-nearest neighbors [37], histogram-based outlier score [38], rotation-based outlier detection [39]; (2) probabilistic: angle-based outlier detection [40], copula-based outlier detection [41]; (3) outlier ensembles: locally selective combination of parallel outlier ensembles [42], lightweight on-line detector of anomalies [43] and isolation forest is the most often used unsupervised anomaly detection algorithm [33]. For the literature review about anomaly detection methods, refer to [44].

**Figure 3:**
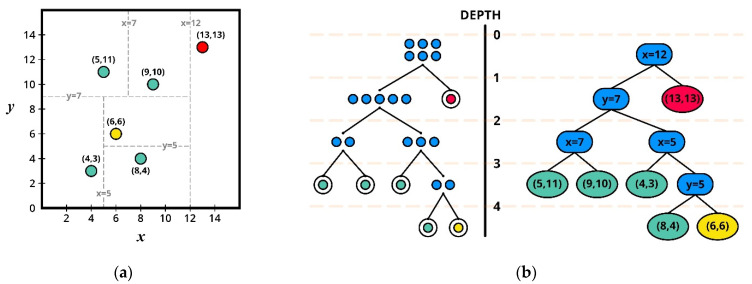
Conceptual illustration of isolation forest: (**a**) isolation operations of data points using randomly constructed binary search tree; (**b**) binary tree with the isolated point.

In this study, an isolation forest was used for the base anomaly detection algorithm. Like random forest, isolation forest consists of multiple binary decision trees (iTrees) trained with different subsamples drawn from original datasets. In the training phase, each decision tree decomposes the data space into two subtrees by the arbitrary values of the randomly chosen feature. The subtree also follows the same procedure until it reaches the stopping condition—(a) if the node contains a single observation or (b) the tree reaches its maximum height. Figure 3 shows the property of the isolation forest. As shown in the figure, a binary search tree (Figure 3b) was randomly constructed to isolate each data point. In Figure 3a, only one split is required to separate the anomalous point (13,13) from the rest of the data, whereas four splits are required to separate the normal point (6,6). As the figure suggests, the abnormal data instance is likely to be closer to the root node than the normal data instance.

As the anomalous data is likely to be closer to the root node, the path length required to reach a data instance would be a proper measure for scoring the anomaly. In the prediction phase, isolation forest measures the degree of the anomaly by measuring the averaging path length from the root to the instance to find the expected path length [45]. More formally, the degree of the anomaly of a data instance d with subsample size n, $S\left(d,n\right)$ can be measured by the following:

(1) $$S\left(d,n\right)=2^{\frac{-E\left(h\left(d\right)\right)}{c\left(n\right)}},$$

where $h\left(d\right)$ is the path length of instance d, $E\left(h\left(d\right)\right)$ is an average value for $h\left(d\right)$ across binary decision trees, and $C\left(n\right)$ represents the average path length given subsample size n, which is calculated as follows:

(2) $$C\left(n\right)=2H\left(n-1\right)-\left(2\left(n-1\right)\right),$$

where $H\left(i\right)$ is the harmonic sum, which is equivalent to the formula ln(i)+0.5772156649. If the anomaly score is close to 1, then d is considered as an anomaly, whereas s is smaller than 0.5, then d is likely to be a normal value. After every data instance’s anomaly score is measured, we can sort them in descending order to find the top anomalies. The detected anomalies may indicate the rapid change of the system status, which shows a significant deviation from the normal baseline.

### 3.3. SHAP on Anomaly Detection

This study aims to explain abnormal data by measuring the contribution of each sensor to this anomaly. Suppose that we want to explain a single input x on a prediction model $f\left(x\right)$. Ideally, if we can represent the single prediction $f\left(x\right)$ by the sum of individual feature importance $\varphi_{i}$, then we can explain the prediction $f\left(x\right)\;$as the following:

(3) $$f\left(x\right)=g\left(x^{\prime }\right)=\varphi_{0}+\varphi_{i}x_{i}^{\prime },$$

where $\varphi_{0}$ is the baseline when all of the input features are missing, $x^{\prime }$ is the binary vector representing whether or not input dimension i is included in the input dimension. This kind of explanation model g is called the additive feature attribution method. Thus, several methods have been proposed to find a good approximation function, g.

SHAP proposed by [26] is a kind of additive feature attribution method. The concept of SHAP originates from Shapley value which was initially proposed by [46]. According to Shapely value, the amount of payoff that a player i gets given a coalition game $\left(v,n\right)$ is:

(4) $$\varphi_{i}\left(v\right)=\sum_{S\subseteq N\backslash \left\{i\right\}}\frac{\left|S\right|!\left(n-\left|S\right|-1\right)!}{n!}\left(v\left(S\cup \left\{i\right\}\right)-v\left(S\right)\right),$$

where *n* is the total players, *S* is a subset of total players that describes a coalition, and *v(S)* is an expected sum of the payoff of coalition *S*. From the above equation, $v\left(S\cup \left\{i\right\}\right)-v\left(S\right)$ is the marginal contribution of the player *i* given the current coalition *S*. Shapely value then can be interpreted as an average of this contribution obtained over every possible permutation of the coalition. The same concept can be applied to machine learning. Given an input instance x for a machine learning model f, if we consider each dimension of x an individual player, this machine learning model can be considered a coalition game whose payoff is determined by the function f. Then, the shapely value $\varphi_{i}\left(f,x\right)$ can be calculated by the following:

(5) $$\varphi_{i}\left(f,x\right)=\sum_{z^{\prime }\subseteq x^{\prime }}\frac{\left|z^{\prime }\right|!\left(n-\left|z^{\prime }\right|-1\right)!}{n!}\left(f\left(z^{\prime }\right)-f\left(z^{\prime }\backslash i\right)\right),$$

where $z^{\prime }$ is a binary vector representing the subset *S* of the features included in the model.

Surprisingly, Lundberg and Lee [26] show that the Shapely value in the above equation is the unique solution to the additive feature attribution model that follows desirable properties (local accuracy, missingness, consistency). To compute the value of the function $f_{x}\left(z^{\prime }\right)$ which contains some missing variables of x, it uses the conditional expectation $E[f\left(z\right)|z_{S}]$ where $z_{S}$ is the set of the variable associated with subset *S*. Of course, the exact calculation of shapely value in Equation (4) is computationally expensive because it has to consider every permutation of input features. However, $E[f\left(z\right)|z_{S}]$ can be further simplified by assuming independence and linearity assumption. In [26], the author proposed the KernelSHAP, which approximates g that can be applied to any machine learning model.

**Figure 4:**
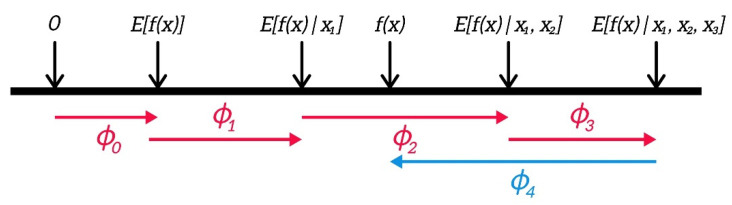
SHAP explanation of machine learning prediction with four variables (adopted from [26]).

Figure 4 illustrates how the SHAP explains the prediction from a machine learning model consisting of four variables. $\varphi_{0}$ is simply the expected value of the function $E\left[f\left(z\right)\right]$ over every dataset, which forms the baseline in feature contribution. Then, by adding each dimension one by one on the conditional term, one can measure this feature’s feature contribution. As SHAP assumes feature independence assumption, the order that the input feature is added is not important. Based on this result, features 1, 2, and 3 increase the prediction from the baseline, whereas feature 4 shows a negative impact. By aggregating Shapely values of every data instance, the global model interpretation is also possible.

In the calculation of SHAP value, treeSHAP algorithm proposed by Lundberg [47] was utilized because it provides a fast and exact feature attribution method by exploiting an ensemble-based decision tree structure. Isolation forest that was chosen for the anomaly detection algorithm for this study is compatible with treeSHAP because it also uses the ensemble-based decision tree in the prediction [47]. Besides, treeSHAP gives specific explainability of black-box information about the local explanation to global understanding on tree-based model machine learning [48]. The ability to easily and precisely generate local explanations using Shapley values across an entire dataset enables the development of a new class of tools for comprehending the isolation forest model’s global behavior to identify the suspicious anomaly event.

**Figure 5:**
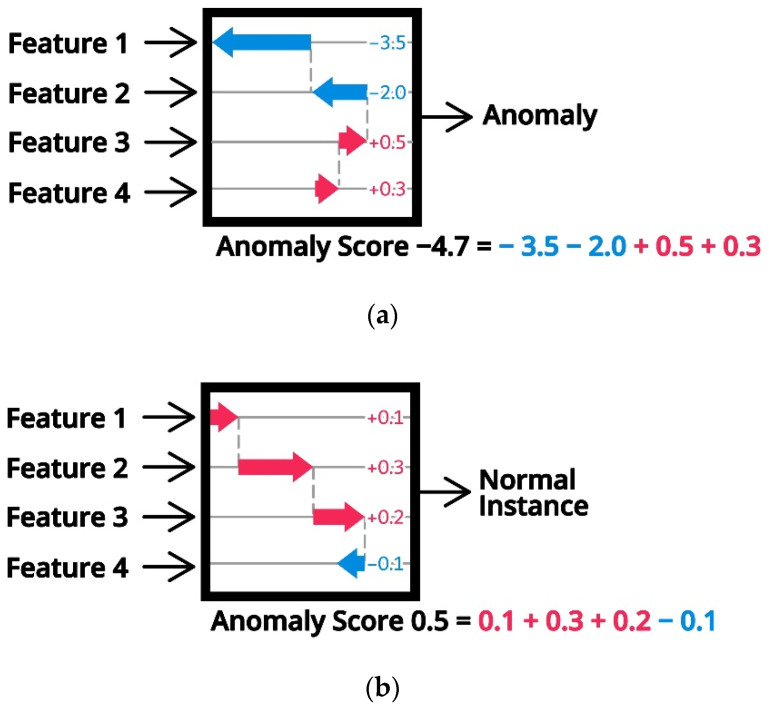
SHAP decomposition between anomalous instance (**a**) and normal instance (**b**).

Figure 5 illustrates an example of SHAP value calculation. Following the property of SHAP value, the sum of each feature’s importance value should be equal to the anomaly score. As the instance with a lower anomaly score is considered an anomaly, the feature with a large negative magnitude may indicate a strong contributor for the anomaly. For example, for anomalous data Figure 5a, features 1 and 2 may be such strong contributors. On the other hand, the SHAP value for the normal instance in Figure 5b may not be associated with such a large negative value. It is also noteworthy that the SHAP value is the unitless measure. This property is helpful because we can compare SHAP values across different features without worrying about the original scale of the feature value.

### 3.4. Hierarchical Clustering

This study performed a hierarchical clustering analysis on the SHAP values of abnormal instances to identify the typical anomaly patterns. The idea is to group similar anomalies which have a similar distribution of SHAP values. Then, each group can be considered an abnormal pattern, providing further insight for the human expert.

Hierarchical clustering is built based on a cluster tree (a dendrogram) representing the data grouped by the similarity between one individual datapoint and another. As the data grouped, groups with similar characteristics will be merged until all groups are merged as one big cluster, which is called agglomerative strategy, or oppositely, from one big cluster that continuously divided into several small clusters based on the heterogeneity of the clusters, which is called divisive strategy.

**Figure 6:**
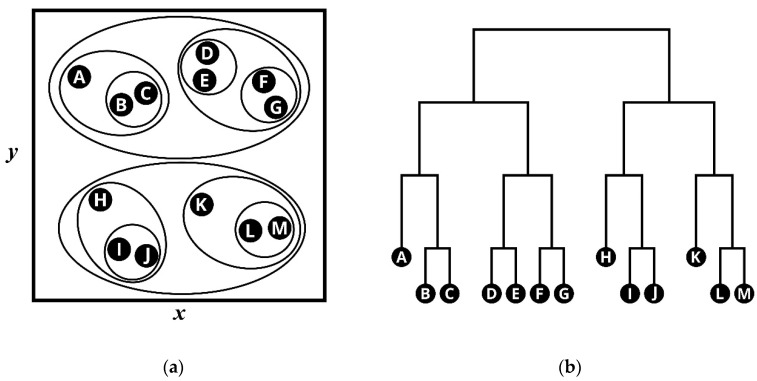
Conceptual illustration of hierarchical clustering: (**a**) nested clusters; (**b**) dendrogram.

In agglomerative clustering, each data point starts as its cluster. Then, the closest pair of clusters is merged based on a distance measure given by the user. This is repeated until all the points are merged into one root cluster [49]. As shown in Figure 6, as the data is grouped and groups are merged, the cluster tree represented in the dendrogram was formed.

The average-linkage method is completed by calculating the distance between a pair of observations, summed up and divided by the total pairs in a cluster, resulting in the average inter-cluster distance. The distance is calculated as follows:

(6) $$d\left(u,v\right)=\sum_{ij}\frac{d\left(u\left[i\right],v\left[j\right]\right)}{\left(\left[u\right]*\left[v\right]\right)},$$

where *u, v* are two clusters, respectively, and *i* and *j* are the data instances belonging to each cluster. From the resulted clustered tree, the determination of the number of clusters was done using the Silhouette method proposed by Peter J. Rousseeuw [50]. This method evaluates the cohesion measure and separation distance between the resulting clusters by the silhouette coefficient. Cohesion measures how closely the data is related to each other in the same cluster while cluster separation measures how separated each cluster is from other clusters. Then the silhouette coefficient is determined by these equations below:

(7) $$s\left(i\right)=\frac{b\left(i\right)-a\left(i\right)}{max\left\{a\left(i\right),b\left(i\right)\right\}},$$

where $s\left(i\right)$ is the silhouette coefficient of sample i that is calculated by comparing the $a\left(i\right)$, as the average distance of i to other samples in the same cluster, to $b\left(i\right)$, as the average distance of all samples of sample i to the other cluster. From Equation (7) the value of the silhouette coefficient range between −1 to 1. Where the more positive value indicates the higher likelihood of i to be assigned in the right cluster. Then the value of K or the best number of clusters is indicated by the higher Silhouette coefficient [51].

## 4. Experiment Result and Discussion

### 4.1. Data Preprocessing

Each data-driven methodology necessitates the use of a representative training dataset. The raw data source, on the other hand, is incomplete. It contains values that are out of control, missing values, redundant variables, and other erroneous data. Without carefully screening the raw data, the resulting model would work poorly on the new data. Thus, multiple preprocessing approaches were used in this analysis to enhance the dataset’s efficiency.

Firstly, out-of-range values that exceeded the acceptable ranges were omitted. These values are often the result of sensor or contact signal failure. Since those are not relevant to the engine’s status, we decided to remove them from the training data collection.

After that, the dataset was compressed by averaging a 10 min interval because the size of the original data with a one-minute interval is too large to train the machine learning model efficiently. Moreover, compared to other vehicles such as cars or aircraft, the vessel engine typically undergoes a slow change in its status; thus, the dataset with too fine granularity may not be practical.

After then, we excluded data gathered when the vessel was stationary since the vessel’s engine is not operational during that time frame. According to expert opinion, we consider areas with an average ground speed greater than 6 knots and an RPM value greater than 70 in this report.

**Figure 7:**
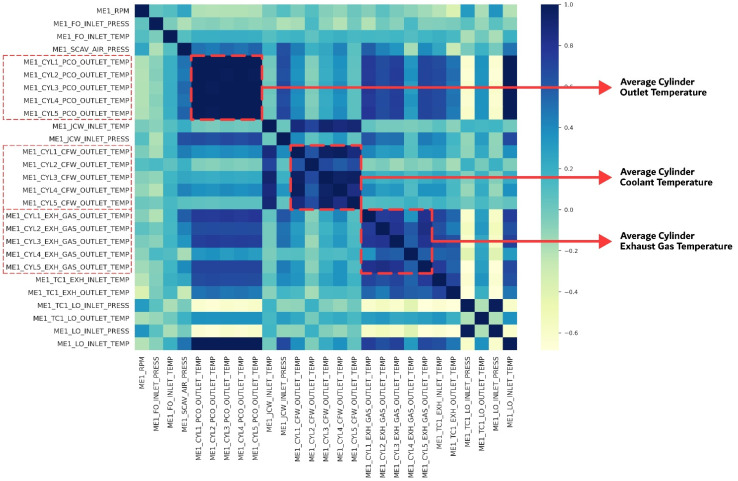
The dimensional reduction process of high correlation features.

**Figure 8:**
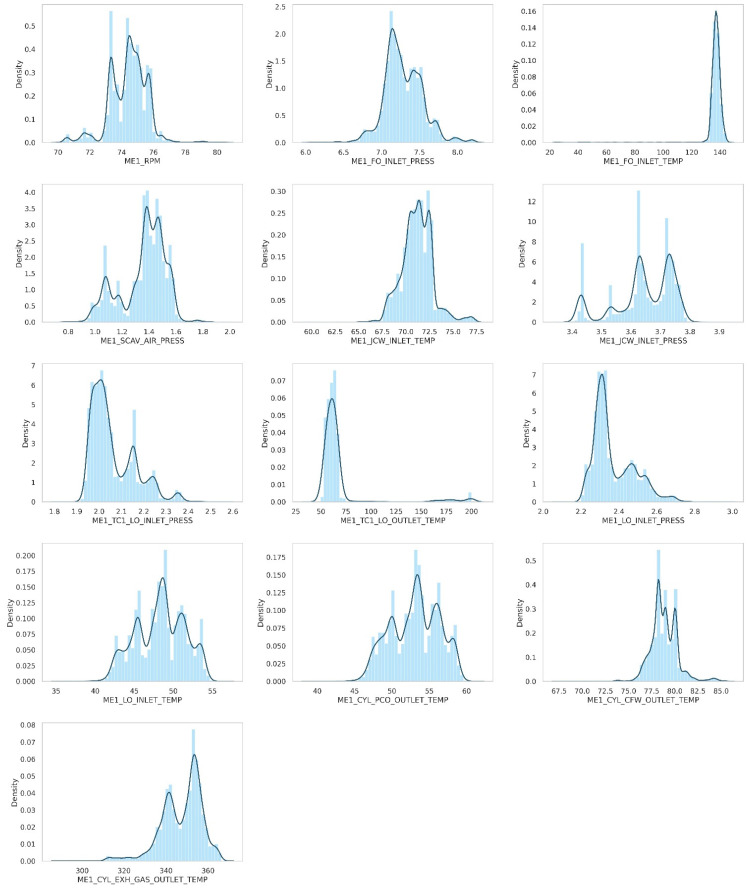
Distribution plot of preprocessed dataset.

In the final stage, rather than using individual sensor values for each cylinder, we used an averaged value since sensor values from five cylinders exhibit a high degree of correlation, suggested by Figure 7. Finally, we obtained the dataset with 13,955 instances. Figure 8 shows the distribution result after preprocessing.

### 4.2. Anomaly Detection Using Isolation Forest

In this step, isolation forest was applied to the preprocessed dataset to find anomalous data instances. Figure 8 shows the distribution of the anomaly score of every data instance. As the histogram has a very thin tail part, it seems that the anomalous data instance is well separated from the rest of the dataset.

**Figure 9:**
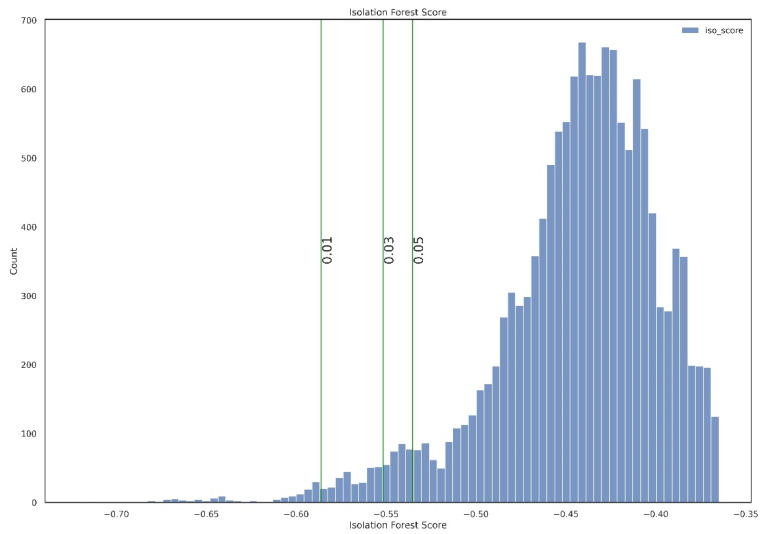
Histogram of anomalous score in different percentages.

We tried three different anomaly score percentiles—1, 3, and 5%—to find good anomaly score criteria. Figure 9 also shows the different anomaly score criteria concerning different percentiles. According to this figure, we concluded to use 1% as our decision criteria because we found a small peak around the 1% vertical line, suggesting that 1% percentile criteria can segment that anomaly group.

### 4.3. Feature Contribution Analysis Using SHAP

**Figure 10:**
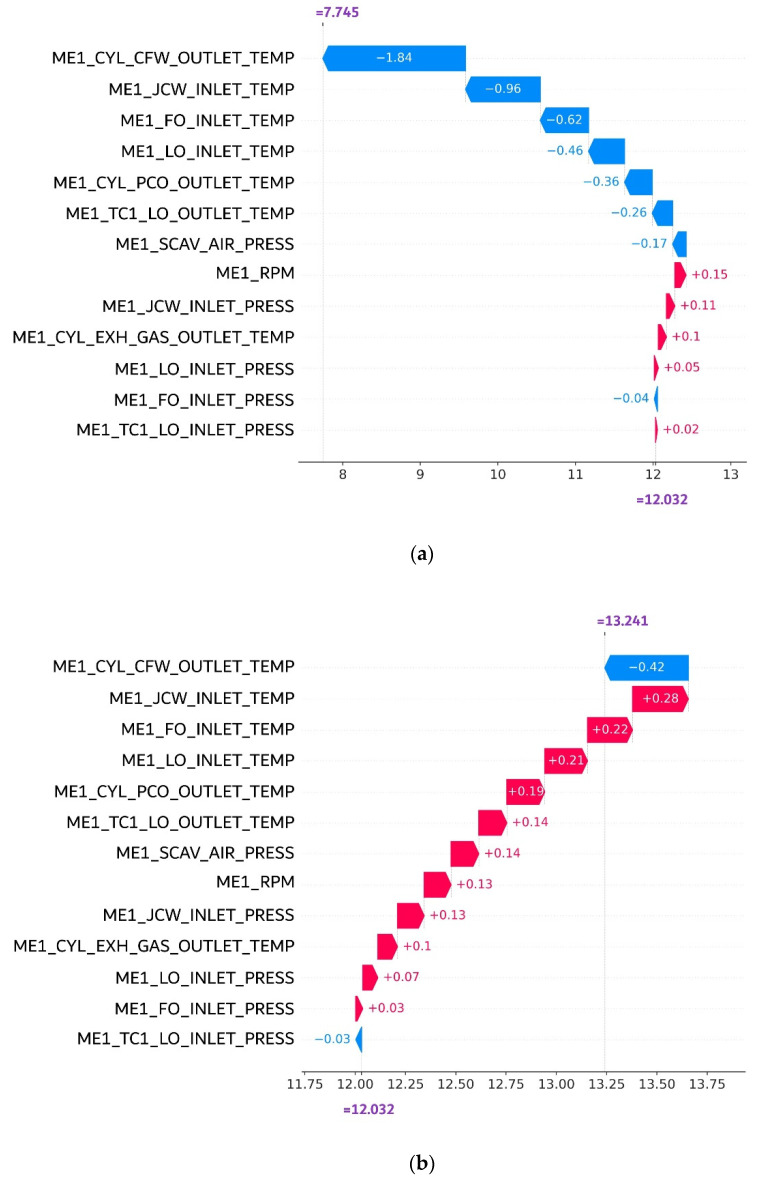
The comparison between SHAP values between two randomly sampled data instances: (**a**) anomaly instances; (**b**) normal instances.

In this step, the isolation forest model was explained by calculating the SHAP value of every dataset instance obtained from the previous stage. Figure 10 compares the SHAP value of an anomalous instance to the normal instance. Both data instances were randomly drawn from two groups (normal and abnormal), respectively. According to the result, the baseline value of the expected anomaly score considering every data instance is 12.032. As shown in Figure 10a, the score of anomalous data instance is 7.745, which is much lower than the baseline. The SHAP value decomposition provides an explanation about which sensor is responsible for this lower anomaly score. According to this figure, the most responsible feature was the ME1_CYL_CFW_OUTLET_TEMP followed by ME1_JCW_INLET_TEMP, ME1_FO_INLET_TEMP, and ME1_LO_INLET_TEMP.

On the other hand, the anomaly score of the randomly drawn normal instance is 13.241, which is slightly larger than the baseline. Its SHAP value decomposition is shown in Figure 10b. As the figure suggests, most of the SHAP values consist of small positive values, except ME1_FO_INLET_PRESS. However, the absolute magnitude of the negative SHAP value was small compared to the anomalous data instance.

In addition to the SHAP value, we also compared the raw sensor value between two data instances in Table 3. As the table suggests, the sensor variables highlighted by the SHAP value in Figure 10a also show a large difference in actual sensor values. This suggests that the SHAP value can explain the anomalous data instance effectively.

**Figure 11:**
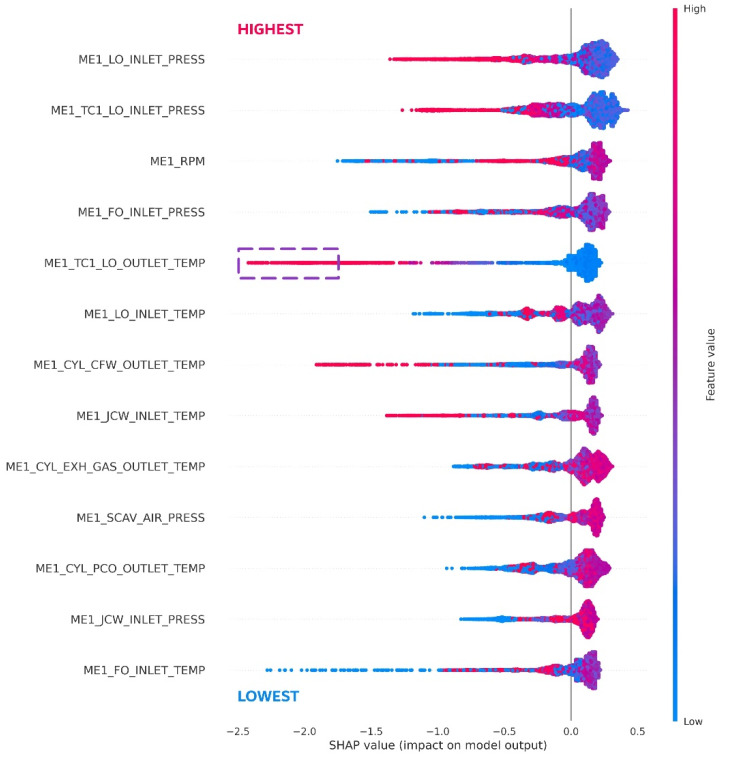
The distribution of SHAP values across entire data instance.

By investigating the distribution of SHAP values over the entire data instance, one can identify the overall trend of each sensor in anomaly detection. Figure 11 shows the global interpretation of SHAP values aggregated over entire data instances. The dot in each feature corresponds to the SHAP values of each data instance. In this figure, the feature is ordered by the average magnitude of the SHAP value, suggesting its distinctiveness power. However, we need to focus on distributing the left tail part because we aim to explain the extreme anomalous data instance. For example, this figure suggests that many extreme anomalies are explained by the main engine turbocharger of lubricant oil outlet temperature (ME1_TC1_LO_OUTLET_TEMP) because extreme negative values are found with this variable. Additionally, we can find that the lower value of ME1_TC1_LO_OUTLET_TEMP would cause the anomaly by referring to its feature value trend. On the contrary, for other sensor variables such as ME1_FO_INLET_PRESS or rpm, anomaly also can be found at both high- and low-value regions.

### 4.4. Anomalous Pattern Detection Using Hierarchical Clustering on Shapely Values

Although the analysis in the previous section enables us to explain the anomaly in both local and global perspectives, the human user may want to explain the anomaly by finding common patterns from the anomalies; that is, grouping the anomalies by their similarity.

**Figure 12:**
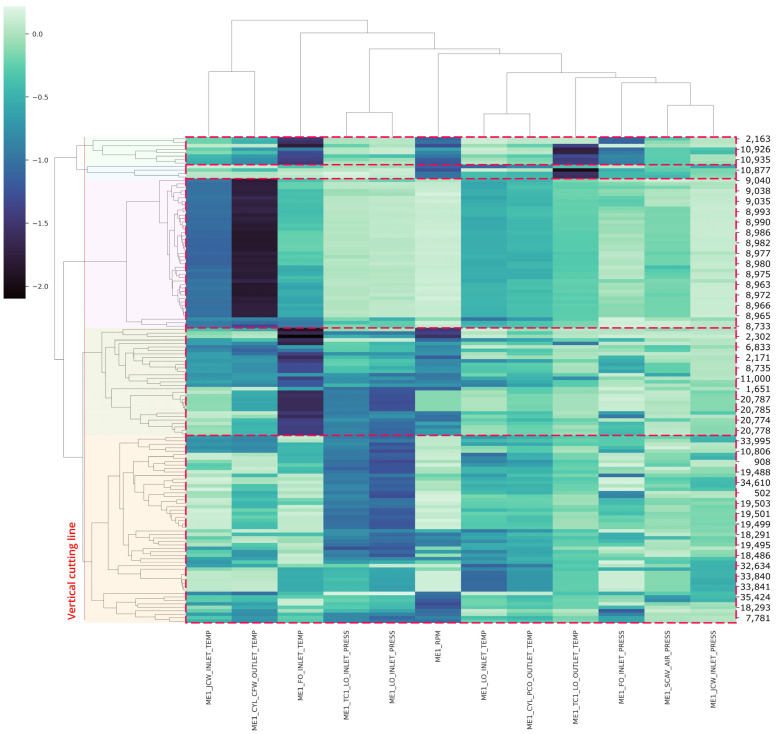
Hierarchical cluster of SHAP anomalous point. Note that the vertical line drawn over the dendrogram determines the subclusters.

To do this, we performed hierarchical clustering to SHAP values of anomalous data instances. The clustering result is shown in Figure 12. As the figure shows, each instance is ordered with similarity to other instances. The dendrogram on the vertical line defines the hierarchical relationship between different sets of data. The height of the dendrogram represents the distance between two subgroups. On the top level of the dendrogram, every data instance belongs to one big cluster. The cluster is further subdivided into sub-clusters until it is decomposed into an individual data instance. In a hierarchical clustering model, cutting a dendrogram at a certain height level results in a set of clusters. Figure 12 illustrates the resulting clusters with the specified threshold. As shown in this figure, the specified vertical line results in a total of five clusters. We also plotted the heatmap of anomalous data to examine the pattern within each cluster further. The feature with a dark color indicates a strong contributor to the anomaly. The figure suggests that anomalous sensors are different across clusters.

**Figure 13:**
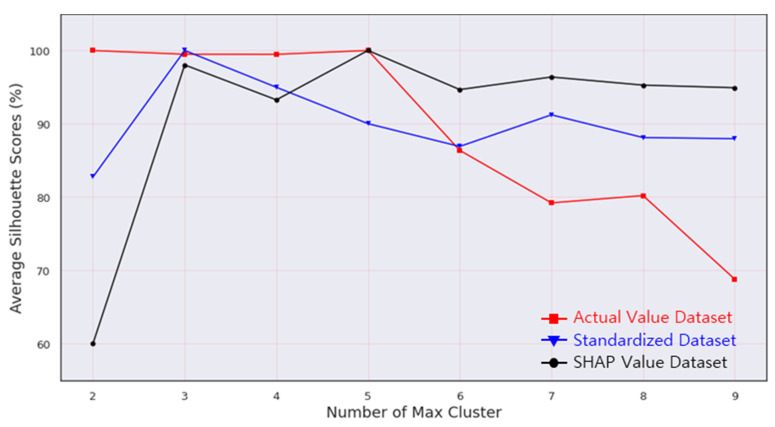
Percentage average silhouette score over clusters.

**Figure 14:**
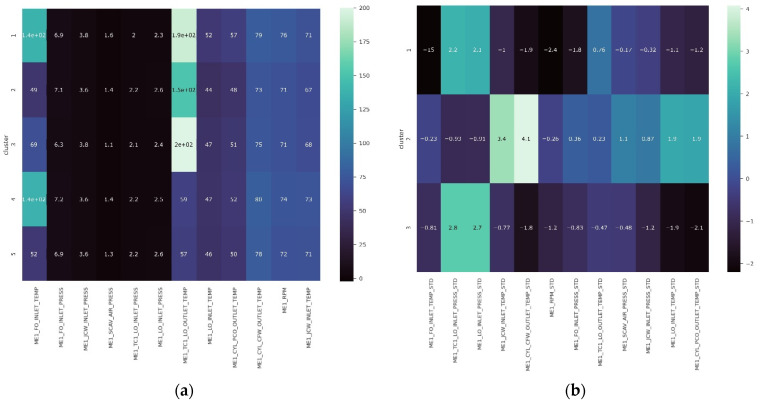

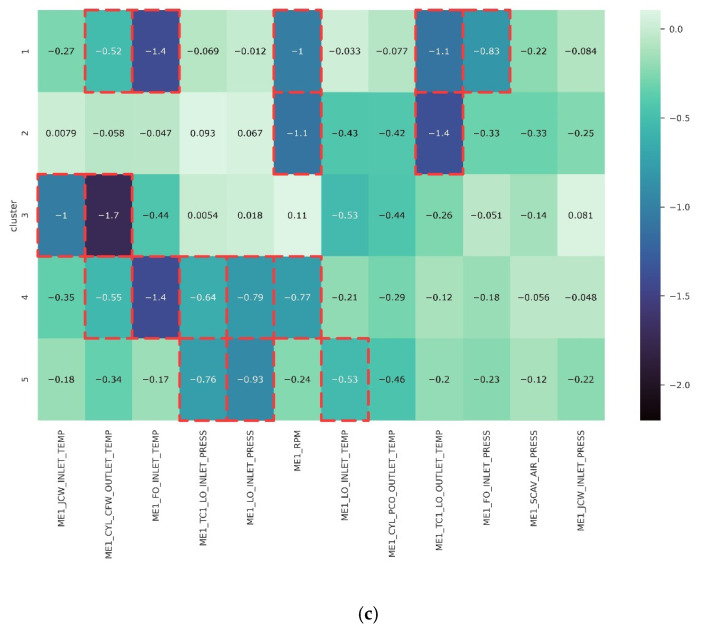
Heatmap comparison of (**a**) actual value, (**b**) standardized value, (**c**) SHAP value.

Following that, the authors compared three experimental methodologies to demonstrate the superior performance of the proposed framework, using hierarchical clustering. Unfortunately, the hierarchical clustering algorithm does not recommend the optimal number of clusters. In this study, a silhouette score was determined to find the optimal cluster number. The result is shown in Figure 13. As the figure shows, the cluster number with the largest silhouette value for actual value, standardized value, and SHAP values were five, three, and five, respectively. Moreover, all results of hierarchical clustering analysis was illustrated in Figure 14 compared to actual value, standardized value, and SHAP value.

The result of hierarchical clustering of actual values is shown in Figure 14a. It is evident that the distribution of each variable was completely dispersed throughout all clusters, as the range of each sensor was on a different scale. The result of the hierarchical clustering of standardized values is given in Figure 14b. This result prompted us to segment the dataset according to the quartile distribution; as a result, the information from this cluster was grouping certain conditions within the same range. For example, regions with a low standardized feature average were clustered together, and vice versa. This standardized clustering method cannot be used to label anomalies, as anomaly conditions can occur in contradictory situations (i.e., some features on high values and some features on low values, however in the same cluster). Following these findings, it was decided that the actual and standardized values clustered did not provide detailed information about what attributes significantly contributed to the anomaly; this fact reinforced the assumption that commonly used approaches were not interpretable.

Furthermore, Figure 14c illustrates this framework, which was initiated using SHAP value transformation. SHAP value provides direct information about which features contribute the most to an anomaly’s contribution to its cluster. For instance, cluster 3 demonstrated two features marked as “ME1_JCW_INLET_TEMP” and “ME1_CYL_CFW_OUTLET_TEMP” that were chosen to be less than −0.5. The lower the SHAP value, the greater the prospective feature contributes to the anomaly situation in this study. Additionally, SHAP values have the same scale distribution across the whole dataset, demonstrating that SHAP’s reliability can outperform all other commonly used approaches. In summary, the detailed advantages that explain the conclusion of SHAP value transformation was overcoming all methods in Table 4.

In detail about explainable suspicious conditions, Table 5 also summarizes the clusters’ anomalous characteristics. To strengthen the explanation of these anomaly conditions, we invited two experts in marine technology and thus, the interpretation of the results of our study can be further confirmed by them. The exact anomaly condition occurred most frequently when multiple features or sensors were in the same cluster condition as explained in the specific Table 5.

Anomalies found in cluster 2 indicate high engine revolutions per minute (RPM) and an abnormally high outlet turbocharger lubricant oil temperature. A potential explanation for this phenomenon is that the engine’s acceleration caused all of the related parameters to change [52]. Besides that, cluster 3 indicates that the vessel’s high performance was continuously utilized, as seen by the high-temperature rate of various sensor components. On the other hand, an explanation for the anomalous parameters in cluster 5 appears to be engine overcooling, in which the engine’s normal operating temperature cannot be reached. Since engine overcooling can be just as damaging as engine overheating, this area warrants further investigation [53]. Additionally, based on the range of Maximum Continuity Rating (MCR) values converted from vessel engine rotation to percentage of power output, the vessel’s condition was “slow ahead” with a risk of failure during deceleration and acceleration especially when the engine was started. This is due to excessive cooling of the inlet temperature and high turbo-charge pressure. Cluster 1 is nearly identical to cluster 4, suggesting that this anomaly occurred as the vessel decelerated prior to reaching the port. This may be proven by the engine’s low rotational speed and intake of fuel oil temperature. However, another component, such as the turbocharger, was operating at a high temperature in cluster 1, whereas in cluster 4, the turbocharger operated at high pressure. According to this study, cluster 1 was explained by deceleration at an unusual temperature rate, while cluster 4 was described by deceleration at an uncommon pressure rate. One possible explanation for this phenomenon is because a marine engine’s intake airflow is erratic [54].

### 4.5. Discussion

In this section, we performed multiple studies to determine the possible causes of the anomalous data point.

**Figure 15:**
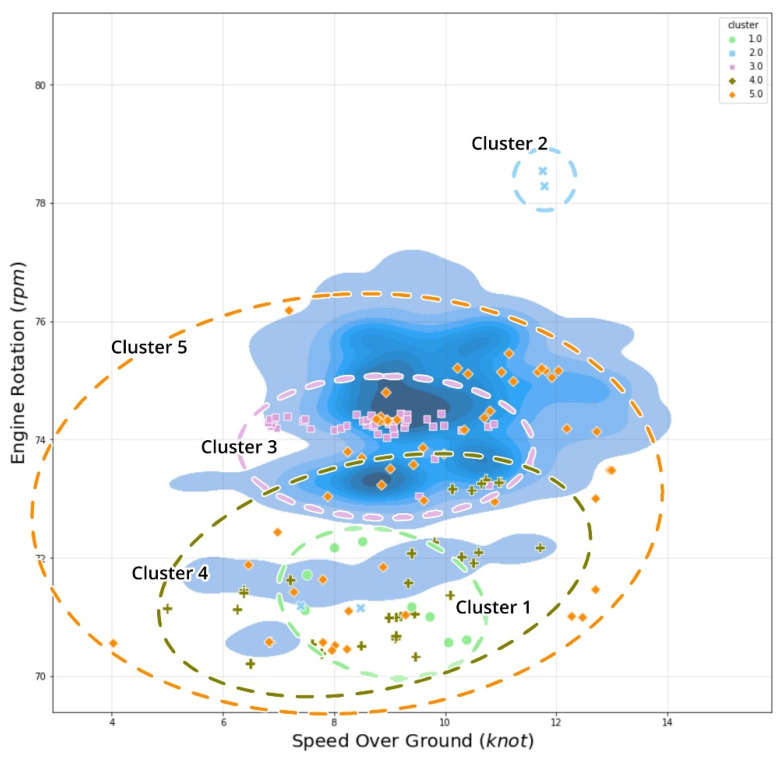
Speed vs. RPM cross plot comparison with cluster distribution.

To begin, we examined the anomalous data point’s position on the vessel’s speed vs. engine rotation graph, as illustrated in Figure 15. Generally, there is a strong correlation between the engine’s rotation per minute and the vessel’s ground speed. This graph demonstrates that cluster 2 has a different distribution than the dataset, being separated only by high-velocity conditions; additionally, it demonstrates that when a vessel performs at high speed overground with a high engine rotation, an anomaly point is labeled as “*overly high performance*” vessel speed occurs. This condition happens mostly within the MCR percentage above 60%.

**Figure 16:**
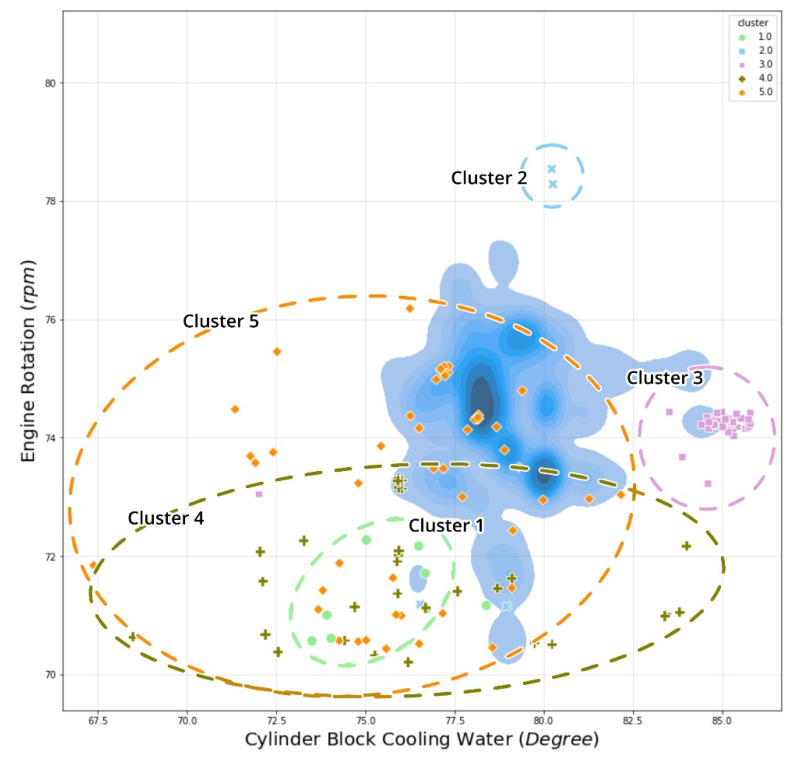
Temperature of cylinder block cooling water vs. RPM cross plot with cluster distribution.

Furthermore, to illustrate the behavior of the vessel temperature, Figure 16 depicts the temperature of the vessel’s cylinder block cooling water vs. engine rotation, which explains why cluster 3 has a relatively high-temperature degree at 58% of MCR within the engine rotation range of about 73–75 rpm. Additionally, the consistent condition of high vessel performance is described since cluster 3 distributions occurred rather frequently under identical behavior conditions. This means that when vessels perform at a high level and generate a lot of heat, an anomaly point labeled “*High performance constantly*” occurred.

**Figure 17:**
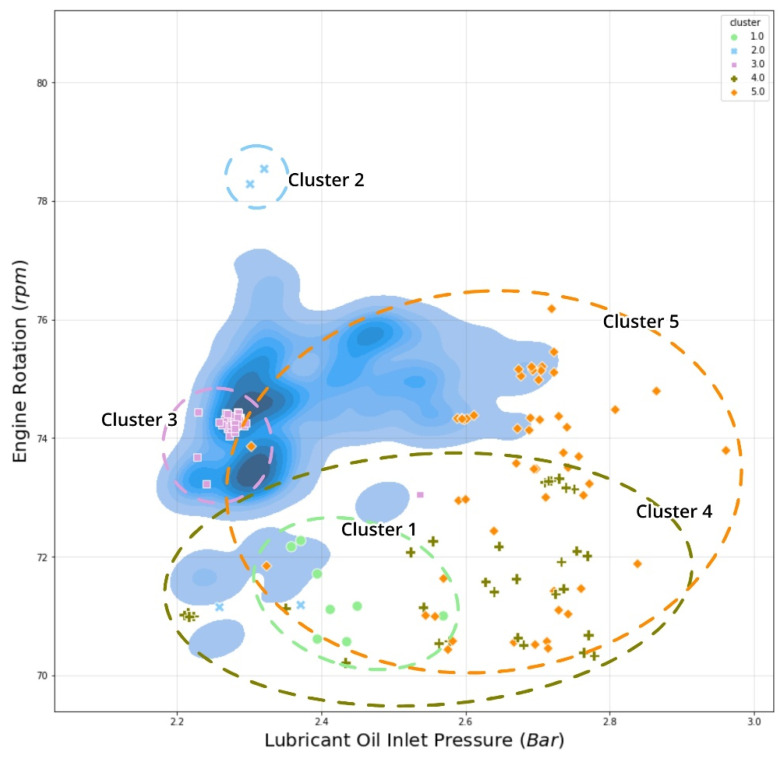
Pressure of lubricant oil inlet vs. RPM cross plot comparison with cluster distribution.

On the other hand, Figure 17 explained the vessel pressure behavior by analyzing lubricant oil pressure vs. engine rotation. This analysis revealed that cluster 1 and cluster 4 had a similar MCR distribution of about 54–58%, which represents vessel velocity deceleration, with a wide range of pressure ranging from 2.2 to 3.0 bar, which was relatively high and, more specifically, for cluster 1, a range of pressure ranging from 2.3 to 2.5 bar. Besides, this explains why the anomaly point of cluster 4 was labeled as “Deceleration; suspicious pressure rate,” which occurred when the engine rotation reduced, but the lubricating oil remained relatively high in pressure. Following all of these findings implies that our anomalous data points might have several causes and necessitates further investigation.

Additionally, as the results using explainable AI frameworks, in detailed investigation, and interpretation outcome, each suspicious condition was technically examined based on the literature review and expert judgments analysis.

**Figure 18:**
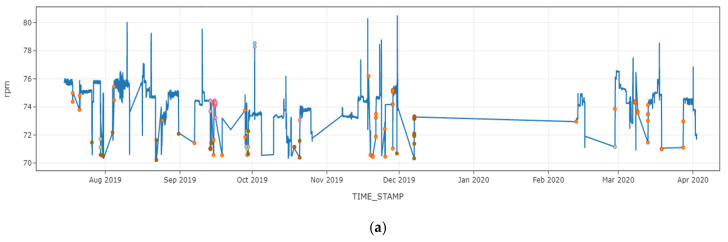

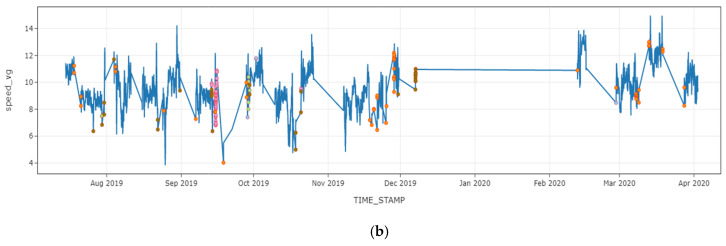
Anomalies plotted over the (**a**) rotation per minute and (**b**) speed over the ground of the vessel.

Additionally, we analyzed the anomalous data point and the time series of (a) rotation per minute (rpm) and (b) speed over ground, as shown in Figure 18. As shown in the figure, the majority of data points in clusters 1, 3, 4, and 5 involved a rapid vessel speed shift. This indicates that the anomaly could be related to acceleration or deceleration, which may result in engine damage.

**Figure 19:**
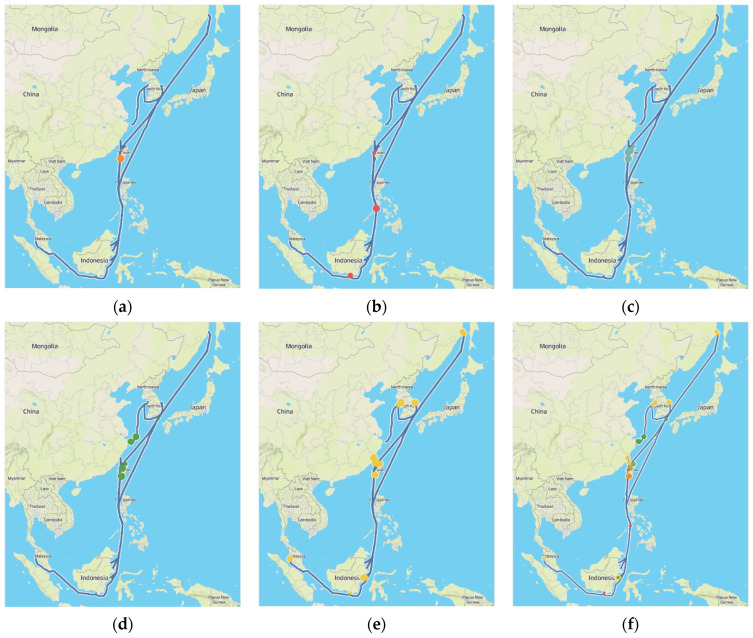
Anomalies plotted over the vessel routes: (**a**) cluster 1; (**b**) cluster 2; (**c**) cluster 3; (**d**) cluster 4; (**e**) cluster 5; (**f**) all cluster.

Finally, as seen in Figure 19, we plotted the anomaly along the vessel’s route. The thin blue line depicts the ships over 10-month navigation direction. As shown in the figure, except for a few instances, the majority of anomalies occurred in adjacent lands. This may be because the vessel is typically pushed at a low speed in coastal waters to avoid collisions, and the engine is run in a different pattern than normal due to regular speed changes. As a result, data collected in coastal waters may be considered anomalous. We believe that this information will aid in the potential identification of the source of engine anomalies encountered during ship service.

## 5. Conclusions

In this study, we proposed a data-driven approach to the condition monitoring of the marine engine. Although several unsupervised methods have existed, the common limitation was the interpretation of the anomaly; they do not explain why the model classifies specific data instances as an anomaly. This study combines explainable AI techniques with anomaly detection algorithm to overcome the limitation above. This study adopts Shapely Additive exPlanation (SHAP), which is theoretically solid and compatible with isolation forest. SHAP enables us to measure the marginal contribution of each sensor variable to an anomaly. Thus, one can easily specify which sensor is responsible for the specific anomaly. Additionally, we proposed to use the hierarchical clustering method on SHAP values to find the typical patterns from anomalies. We validated our method using the dataset obtained from the actual cargo vessel. The results proved that the detected and clustered anomalies could be investigated further to their contribution value to the final decision of anomaly detection.

One of the possible future research directions is to apply our method to the labeled dataset. For example, the same procedure can be applied to explain the classification model that learns the simulated run-to-failure data generated by the artificial simulator. SHAP can also explain the prediction by specifying some of the engine sensors responsible for a fault prediction. The interpretation obtained from the simulation model could then further be utilized in the actual operating environment.

## Figures and Tables

**Table 1:** Vessel specification.

Specification	
Length overall	269.36 m
Length between perpendiculars	259.00 m
Breadth	43.00 m
Depth	23.80 m
Draught	17.30 m
Deadweight	152.517 metric t

**Table 2:** Data features list.

Feature Name	Description
TIME_STAMP	A time when the data is recorded
ME1_FO_TEMP_INLET	Temperature of fuel oil
ME1_RPM	Engine rotation per minute (RPM)
ME1_FO_INLET_PRESS	Inlet pressure of fuel oil
ME1_FO_INLET_TEMP	Inlet temperature of fuel oil
ME1_SCAV_AIR_PRESS	Pressure of scavenging air
ME1_JCW_INLET_TEMP	Inlet temperature of jacket cooling water
ME1_JCW_INLET_PRESS	Inlet pressure of jacket cooling water
ME1_TC1_EXH_INLET_TEMP	Inlet temperature of exhaust gas of turbocharger
ME1_TC1_EXH_OUTLET_TEMP	Outlet temperature of exhaust gas of turbocharger
ME1_TC1_LO_INLET_PRESS	Inlet pressure of lubricant oil in turbocharger
ME1_TC1_LO_OUTLET_TEMP	Outlet temperature of lubricant oil in turbocharger
ME1_LO_INLET_PRESS	Inlet pressure of lubricant oil
ME1_LO_INLET_TEMP	Inlet temperature of lubricant oil
ME1_CYL [1~5]_PCO_OUTLET_TEMP	Outlet temperature of cylinder piston cooling oil (cylinders 1 to 5)
ME1_CYL [1~5]_CFW_OUTLET_TEMP	Outlet temperature of cylinder block cooling water (cylinders 1 to 5)
ME1_CYL [1~5]_EXH_GAS_OUTLET_TEMP	Outlet temperature of exhaust gas (cylinders 1 to 5)

**Table 3:** Comparison of actual values between two data instances in Figure 10.

Feature Name	Anomalous Instances	Normal Instances
	12 September 2019 (7:10 KST)	21 October 2019 (13:50 KST)
ME1_RPM	74.279	73.644
ME1_FO_INLET_PRESS	7.473	6.901
ME1_FO_INLET_TEMP	142.913	138.045
ME1_SCAV_AIR_PRESS	1.576	1.343
ME1_JCW_INLET_TEMP	76.800	71.670
ME1_JCW_INLET_PRESS	3.727	3.646
ME1_TC1_LO_INLET_PRESS	1.973	2.048
ME1_TC1_LO_OUTLET_TEMP	68.908	57.647
ME1_LO_INLET_PRESS	2.279	2.363
ME1_LO_INLET_TEMP	54.290	45.948
ME1_CYL_PCO_OUTLET_TEMP	59.117	50.787
ME1_CYL_CFW_OUTLET_TEMP	84.867	78.983
ME1_CYL_EXH_GAS_OUTLET_TEMP	354.501	341.918

**Table 4:** Comparison of framework performance based on the dataset.

Parameters	Actual Value Dataset (a)	Standardized Dataset (b)	SHAP Value Dataset (c)
Feature importance	✗	✗	✓
Instance scoring	✗	✗	✓
Detect anomaly on the same subquartile	✗	✓	✓
Detect anomaly on contradictory quartile region	✗	✗	✓
Heatmap result segmentation	✓	✓	✓

**Table 5:** Explanation of each cluster obtained from Figure 14.

Clusters	Anomalous Features	Description	Vessel Status
Cluster 1	ME1_CYL_CFW_OUTLET_TEMP	Intermediate to a low temperature of cylinder block cooling water	(***Deceleration; suspicious temperature rate***) Docking to the port MCR 55–57% Deceleration/acceleration
	ME1_FO_INLET_TEMP	Low inlet temperature of fuel oil	
	ME1_RPM	Low engine rotation per minute (RPM)	
	ME1_TC1_LO_OUTLET_TEMP	Extreme high outlet turbocharger temperature of lubricant oil	
	ME1_FO_INLET_PRESS	Extreme low inlet pressure of fuel oil	
Cluster 2	ME1_RPM	High engine rotation per minute (RPM)	(***Overly high performance***) High cruise speed (>10 knots) MCR > 60%
	ME1_TC1_LO_OUTLET_TEMP	Extreme high outlet turbocharger Temperature of lubricant oil	
Cluster 3	ME1_JCW_INLET_TEMP	High inlet temperature of jacket cooling water	(***High performance constantly***) Cruise speed MCR 58%
	ME1_CYL_CFW_OUTLET_TEMP	High temperature of cylinder block cooling water	
	ME1_LO_INLET_TEMP	High inlet temperature of lubricant oil	
Cluster 4	ME1_CYL_CFW_OUTLET_TEMP	Intermediate to a low temperature of cylinder block cooling water	(***Deceleration; suspicious pressure rate***) Docking to the port or Slow ahead MCR 54–58% Deceleration/acceleration
	ME1_FO_INLET_TEMP	Extreme low inlet temperature of fuel oil	
	ME1_TC1_LO_INLET_PRESS	Intermediate to high outlet turbocharger pressure of lubricant oil	
	ME1_LO_INLET_PRESS	Intermediate to high inlet pressure of lubricant oil	
	ME1_RPM	Low engine rotation per minute (RPM)	
Cluster 5	ME1_TC1_LO_INLET_PRESS	High turbocharger inlet pressure of lubricant oil	(***Overcooling engine***) Slow ahead MCR 55–60% Possibility engine start
	ME1_LO_INLET_PRESS	Intermediate to high inlet pressure of lubricant oil	
	ME1_LO_INLET_TEMP	Low to extreme low inlet temperature of lubricant oil	
